# Supplementary material for: Catalytic ozonation mechanism over M1-N3C1 active sites
Source: Nat Commun. 2023 Nov 2;14:7011. doi: 10.1038/s41467-023-42853-8 (PMC10622452; doi:10.1038/s41467-023-42853-8)
Supplement: Supplementary file 1 — Supplementary Information [file 41467_2023_42853_MOESM1_ESM.pdf]

Supplementary Information

for

Catalytic ozonation mechanism over M_1-N_3C_1 active sites

Dingren Ma[1], Qiyu Lian[1], Yexing Zhang[1], Yajing Huang[1], Xinyi Guan[1], Qiwen Liang[1], Chun He[1], Dehua Xia[1]*, Shengwei Liu[1]*, and Jiaguo Yu[2]*

[1] School of Environmental Science and Engineering, Guangdong Provincial Key Laboratory of Environmental Pollution Control and Remediation Technology, Sun Yat-sen University, Guangzhou 510275, China

[2] Laboratory of Solar Fuel, Faculty of Materials Science and Chemistry, China University of Geosciences, 68 Jincheng Street, Wuhan 430078, China

* These authors jointly supervised this work: Dehua Xia (xiadehua3@mail.sysu.edu.cn); Shengwei Liu (liushw6@mail.sysu.edu.cn); Jiaguo Yu (yujiaguo93@cug.edu.cn).

Table of Contents

Supplementary Fig. 1. **Preparation of the MNC catalysts.** 4

Supplementary Fig. 2. **Morphology of the MNC catalysts.** 5

Supplementary Fig. 3. **Structure of the MNC catalysts.** 6

Supplementary Fig. 4. **Morphology of the MNC catalysts.** 7

Supplementary Fig. 5. **Morphology of the NC catalyst.** 8

Supplementary Fig. 6. **Raman spectra.** 9

Supplementary Fig. 7. **Composition of the MnNC.** 10

Supplementary Fig. 8. **Composition of the FeNC.** 11

Supplementary Fig. 9. **Composition of the CoNC.** 12

Supplementary Fig. 10. **Composition of the NiNC.** 13

Supplementary Fig. 11. **Fourier transform infrared spectra.** 14

Supplementary Fig. 12. **Wavelet transform.** 15

Supplementary Fig. 13. **Theoretical models of the MNC catalysts.** 16

Supplementary Fig. 14. **Theoretical models of the M_1-N_4 units.** 17

Supplementary Fig. 15. **Partially density of states.** 18

Supplementary Fig. 16. **The charge distribution of the M_1-N_3C_1 units.** 19

Supplementary Fig. 17. **The charge distribution of the M_1-N_4 units.** 20

Supplementary Fig. 18. **The *d*-band centers.** 21

Supplementary Fig. 19. **The regeneration ability of the CoNC.** 22

Supplementary Fig. 20. **Exhaust gas concentration after catalytic ozonation.** 23

Supplementary Fig. 21. **Ozone conversion efficiency in catalytic ozonation.** 24

Supplementary Fig. 22. **Methyl mercaptan dynamic degradation tests in the air.** 25

Supplementary Fig. 23. **Exhaust gas concentration after methyl mercaptan dynamic degradation tests in the air.** 26

Supplementary Fig. 24. **Exhaust gas concentration after methyl mercaptan dynamic degradation tests in the air.** 27

Supplementary Fig. 25. **Surface composition of the reacted CoNC.** 28

Supplementary Fig. 26. **Catalytic ozonation tests under different relative humidity conditions.** 29

Supplementary Fig. 27. **Catalytic ozone decomposition tests under different relative humidity conditions.** 30

Supplementary Fig. 28. **Catalytic ozonation tests under different catalyst dosages.** 31

Supplementary Fig. 29. **The mass activity in the tests of catalytic ozonation.** 32
Supplementary Fig. 30. **Catalytic ozonation tests.**.. 33
Supplementary Fig. 31. **Long-term test of catalytic ozonation.**... 34
Supplementary Fig. 32. **Morphology of the used CoNC.** 35
Supplementary Fig. 33. **Composition of the used CoNC.**... 36
Supplementary Fig. 34. **Reaction of ozone on the surface of the MNC catalysts.**.............. 37
Supplementary Fig. 35. **Complexes on the surface of the MNC catalysts in the air.**......... 38
Supplementary Fig. 36. **Reactivity of MNC catalyst surface complexes.** 39
Supplementary Fig. 37. **Reaction process of methyl mercaptan on the surface of the MNC catalysts.** 40
Supplementary Fig. 38. **Reaction process of catalytic ozonation.**....................................... 41
Supplementary Fig. 39. **Species accumulation on the CoNC.**.. 42
Supplementary Fig. 40. **Reaction process of catalytic ozonation on the surface of the NC.** 43
Supplementary Fig. 41. **Reactions between ozone molecules and single metal atoms.**...... 44
Supplementary Fig. 42. **Reaction between ozone molecule and C atom.**........................... 45
Supplementary Fig. 43. **Theoretical models of the M−*O complexes.**.............................. 46
Supplementary Fig. 44. **Schematic diagram of the experimental device.**.......................... 47
Supplementary Table 1. Textural properties of the MNC catalysts. 48
Supplementary Table 2. The elemental content and state percentages of the MNC catalysts. 49
Supplementary Table 3. Extended X-ray absorption fine structure fitting data for Co center at the Co K-edge.. 50
Supplementary Table 4. Identified conversion products of methyl mercaptan in the air and ozone atmosphere by PTR-TOF-MS... 51
Supplementary Table 5. The elemental content and state percentages of samples. 52
Supplementary Table 6. Detected infrared bands over samples.. 53
Supplementary Table 7. Optimize the adsorption energy corresponding to the structure of the resting point in the molecular adsorption process of the MNC catalysts. 54
Supplementary Table 8. Optimize the free energy corresponding to the structure of the resting point in the ozone decomposition process of the MNC catalysts.. 55

Supplementary Figures

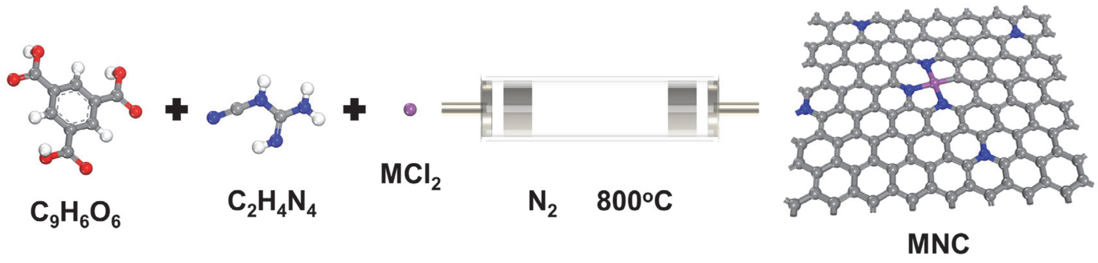

Supplementary Fig. 1. **Preparation of the MNC catalysts.** Schematic of the preparation strategy for the MNC catalysts.

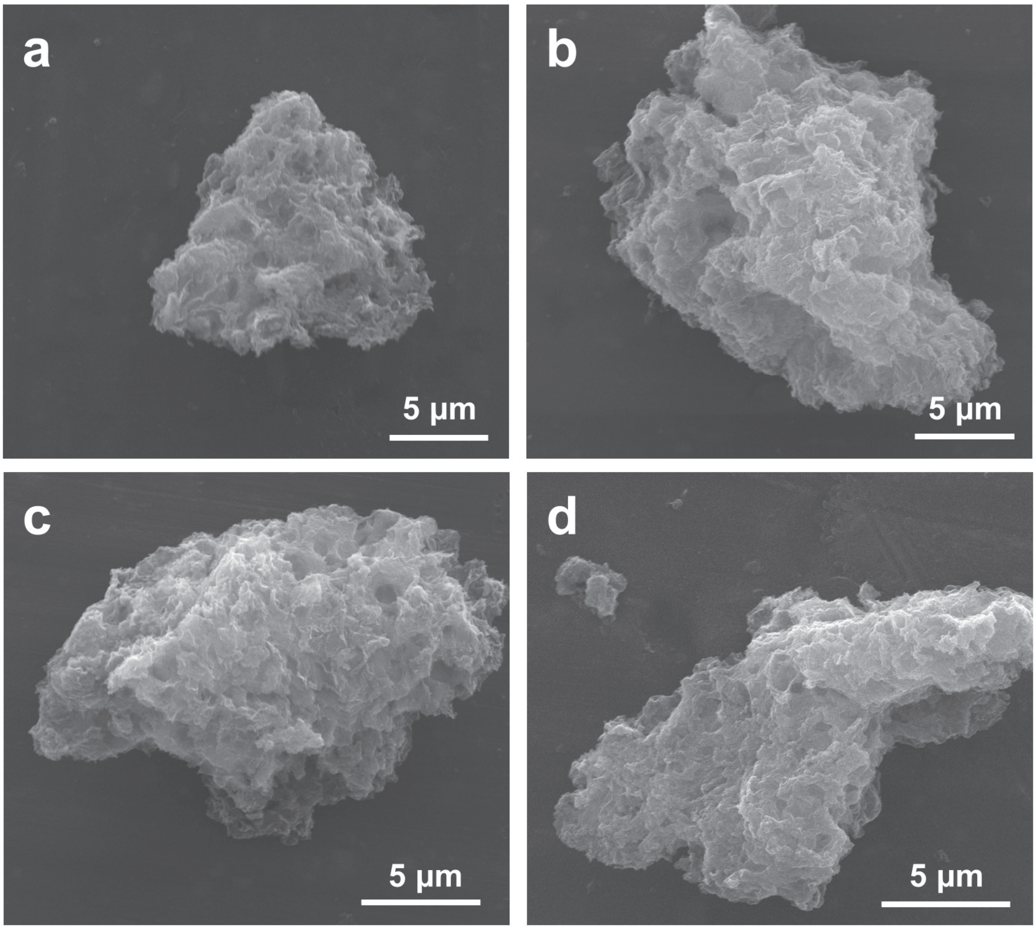

Supplementary Fig. 2. **Morphology of the MNC catalysts.** Scanning electron microscope (SEM) images of the MNC catalysts (**a−d** MnNC, FeNC, CoNC, and NiNC).

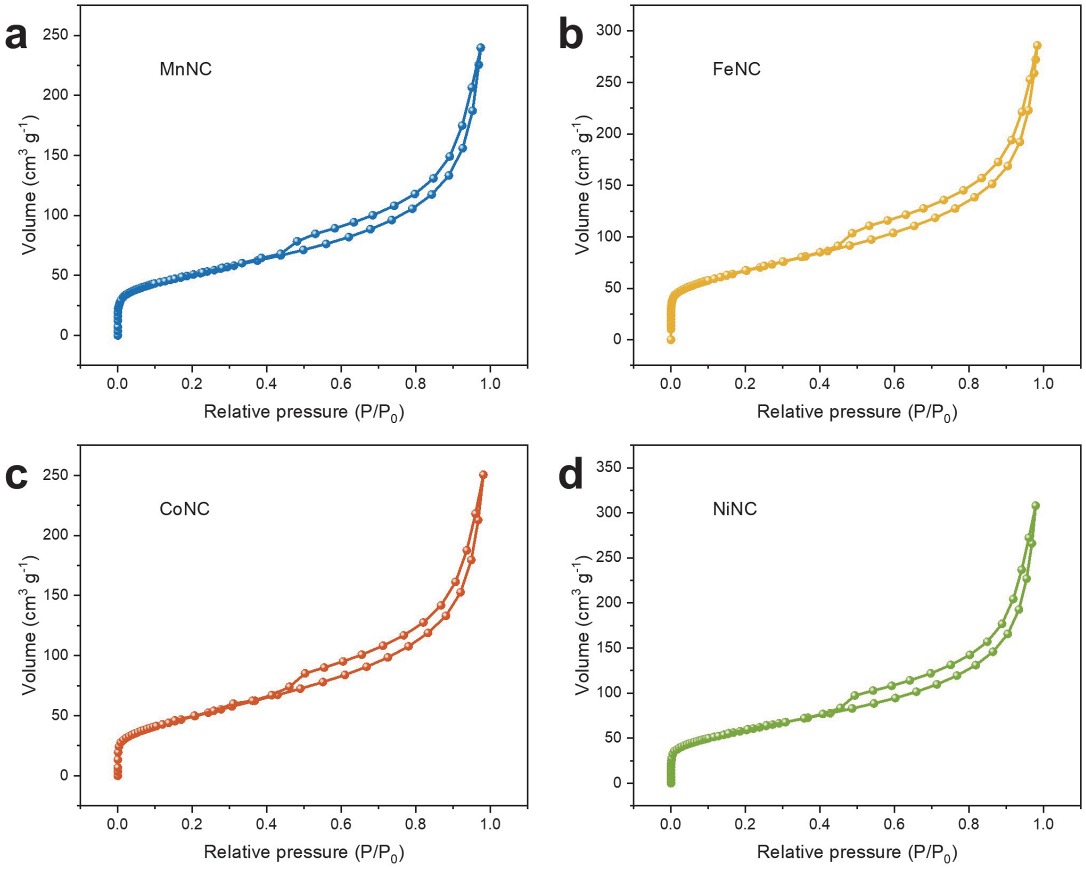

Supplementary Fig. 3. **Structure of the MNC catalysts.** N_2 absorption-desorption isotherms of the MNC catalysts (**a−d** MnNC, FeNC, CoNC, and NiNC).

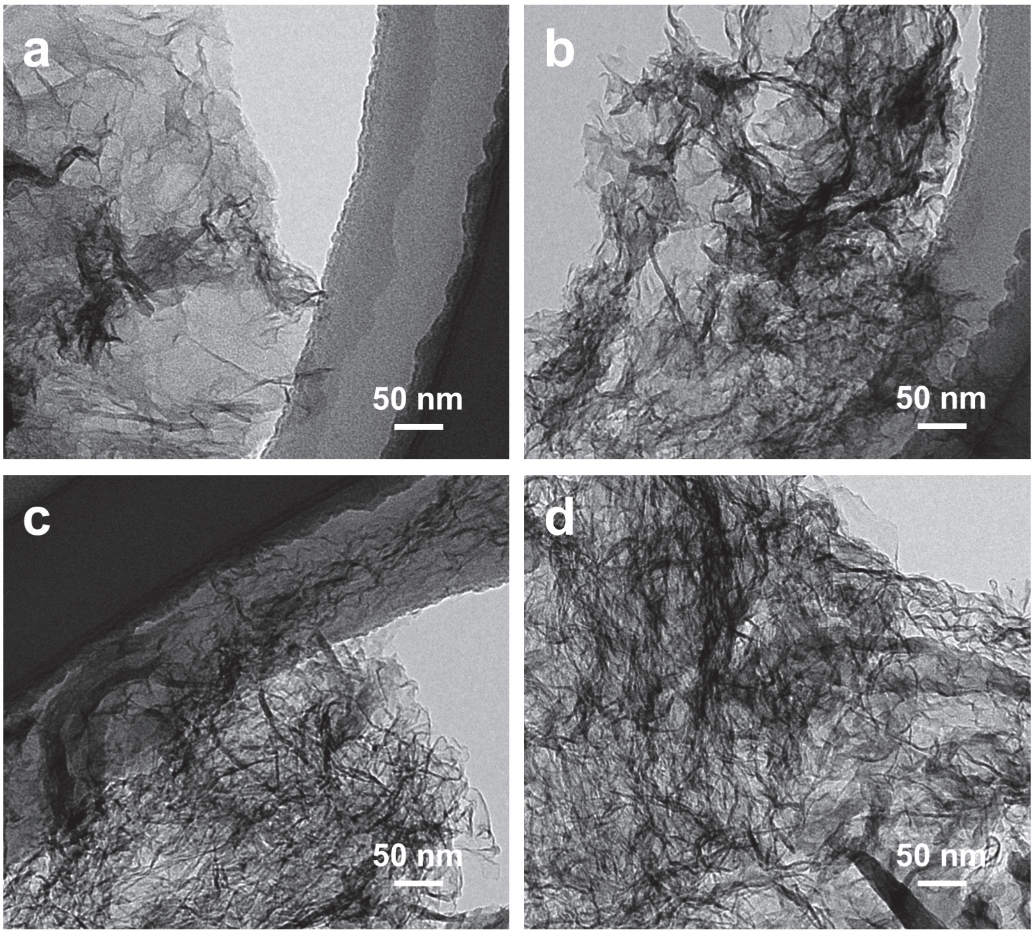

Supplementary Fig. 4. **Morphology of the MNC catalysts.** Transmission electron microscope (TEM) images of the MNC catalysts (**a−d** MnNC, FeNC, CoNC, and NiNC).

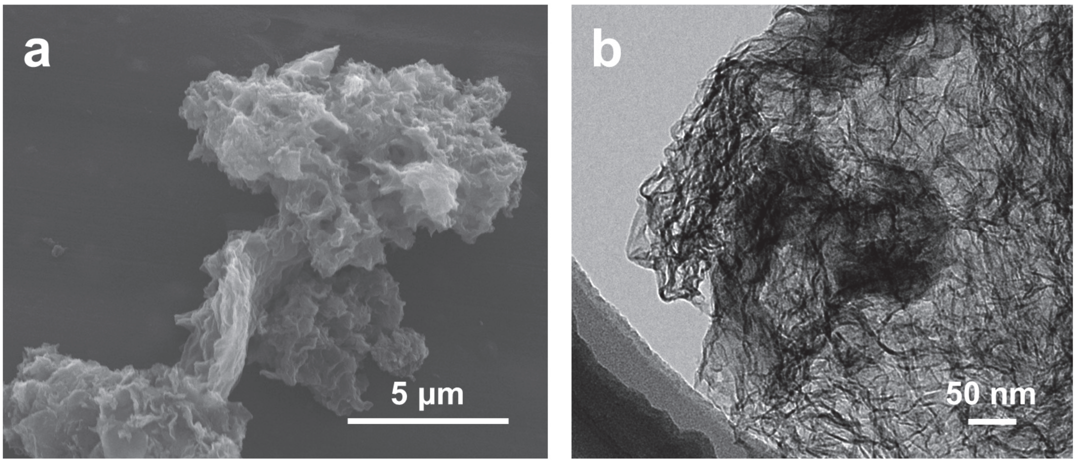

Supplementary Fig. 5. **Morphology of the NC catalyst.** Scanning electron microscope (**a** SEM) and transmission electron microscope (**b** TEM) images of the NC.

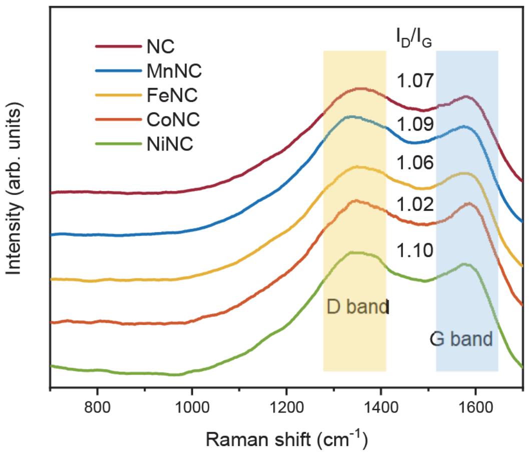

Supplementary Fig. 6. **Raman spectra.** Raman spectra of the NC and MNC catalysts.

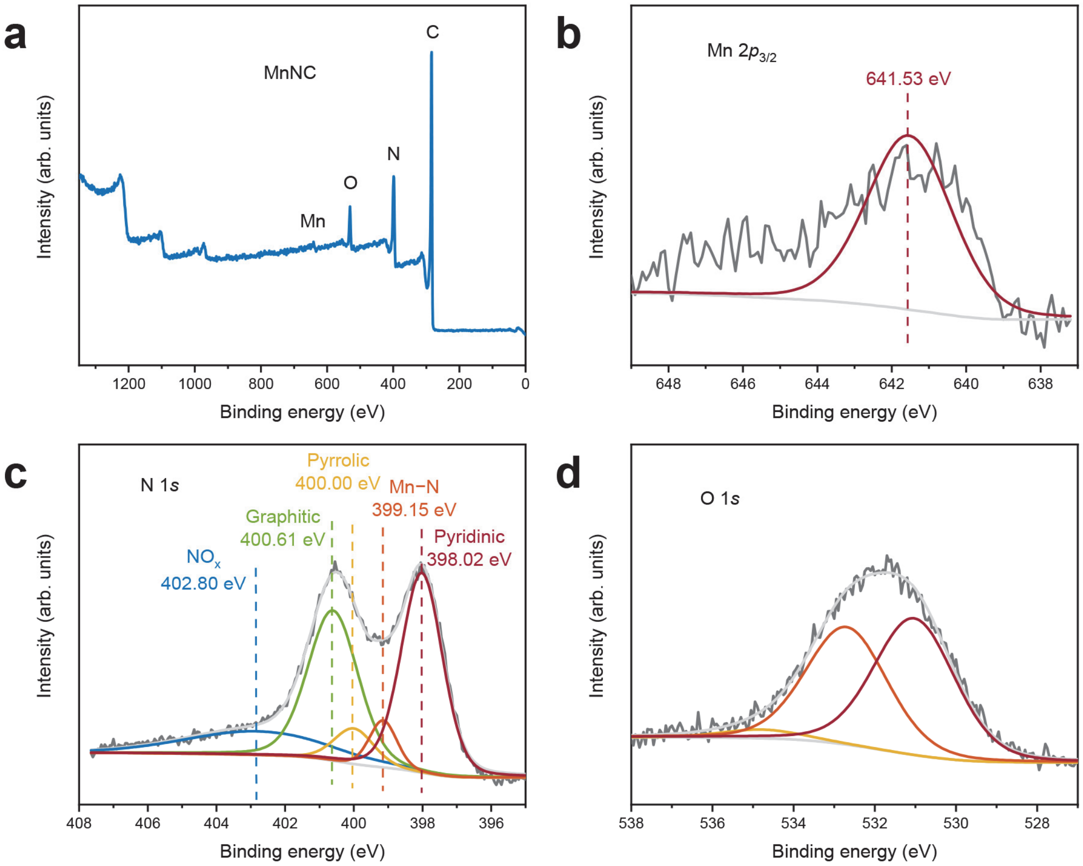

Supplementary Fig. 7. **Composition of the MnNC.** X-ray photoelectron spectroscopy (XPS) of the MnNC (**a** survey spectrum. **b** Mn 2$p_{3/2}$ spectrum. **c** N 1*s* spectrum. **d** O 1*s* spectrum.).

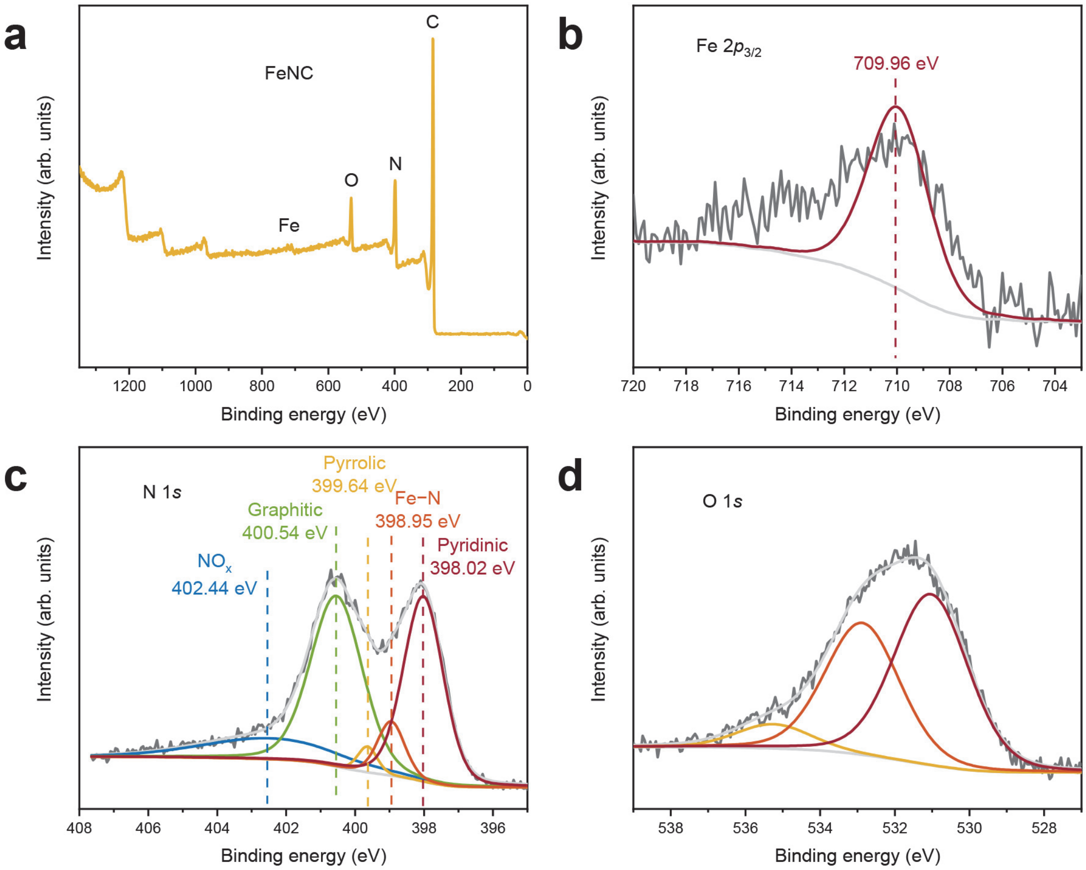

Supplementary Fig. 8. **Composition of the FeNC.** X-ray photoelectron spectroscopy (XPS) of the FeNC (**a** survey spectrum. **b** Fe $2p_{3/2}$ spectrum. **c** N 1*s* spectrum. **d** O 1*s* spectrum.).

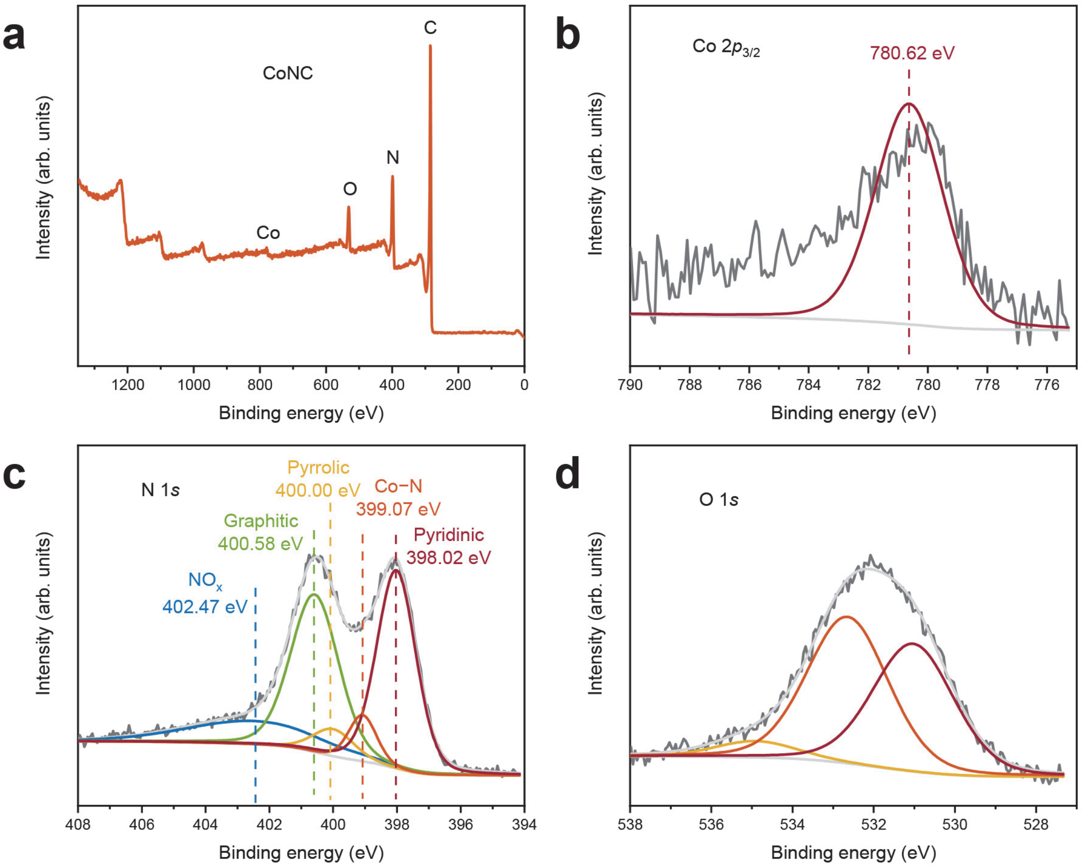

Supplementary Fig. 9. **Composition of the CoNC.** X-ray photoelectron spectroscopy (XPS) of the CoNC (**a** survey spectrum. **b** Co 2$p_{3/2}$ spectrum. **c** N 1*s* spectrum. **d** O 1*s* spectrum.).

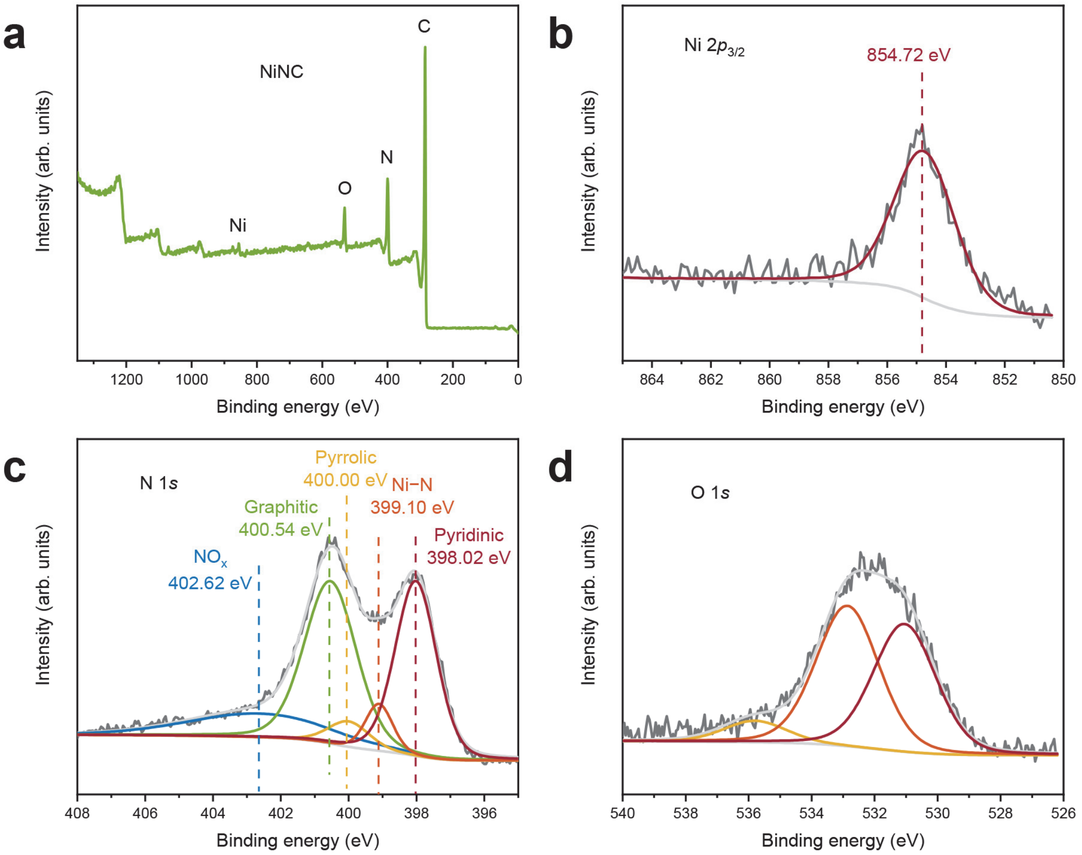

Supplementary Fig. 10. **Composition of the NiNC.** X-ray photoelectron spectroscopy (XPS) of the NiNC (**a** survey spectrum. **b** Ni 2$p_{3/2}$ spectrum. **c** N 1*s* spectrum. **d** O 1*s* spectrum.).

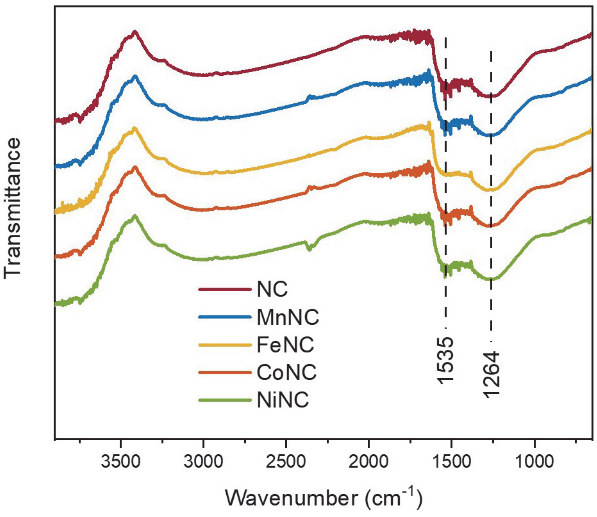

Supplementary Fig. 11. **Fourier transform infrared spectra.** Fourier transform infrared (FTIR) spectra of the NC and MNC catalysts.

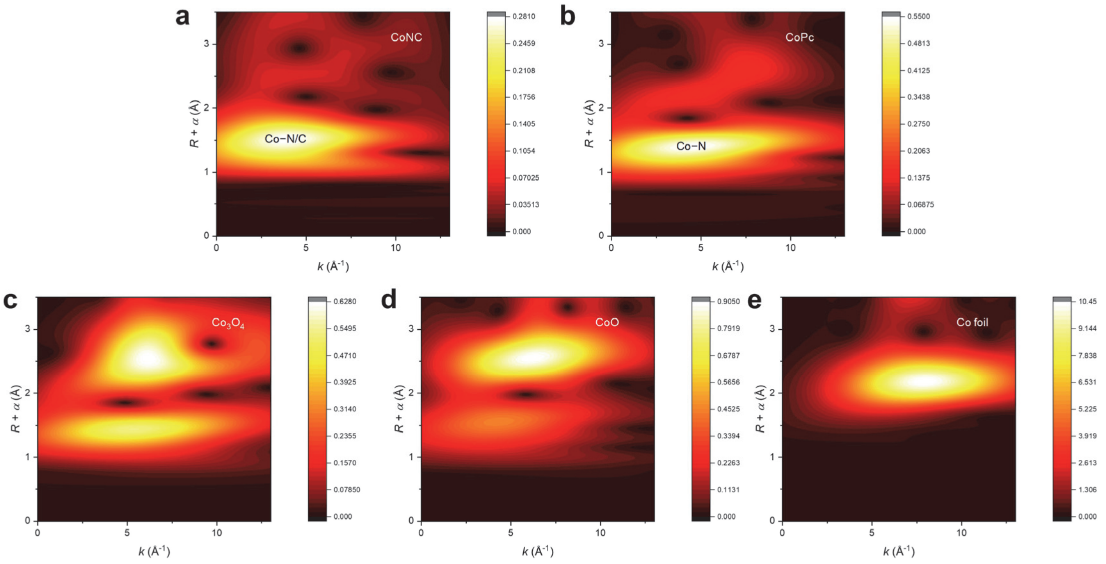

Supplementary Fig. 12. **Wavelet transform.** Wavelet transform (WT) of the CoNC (**a**), CoPc (**b**), Co_3O_4 (**c**), CoO (**d**), and Co-foil (**e**).

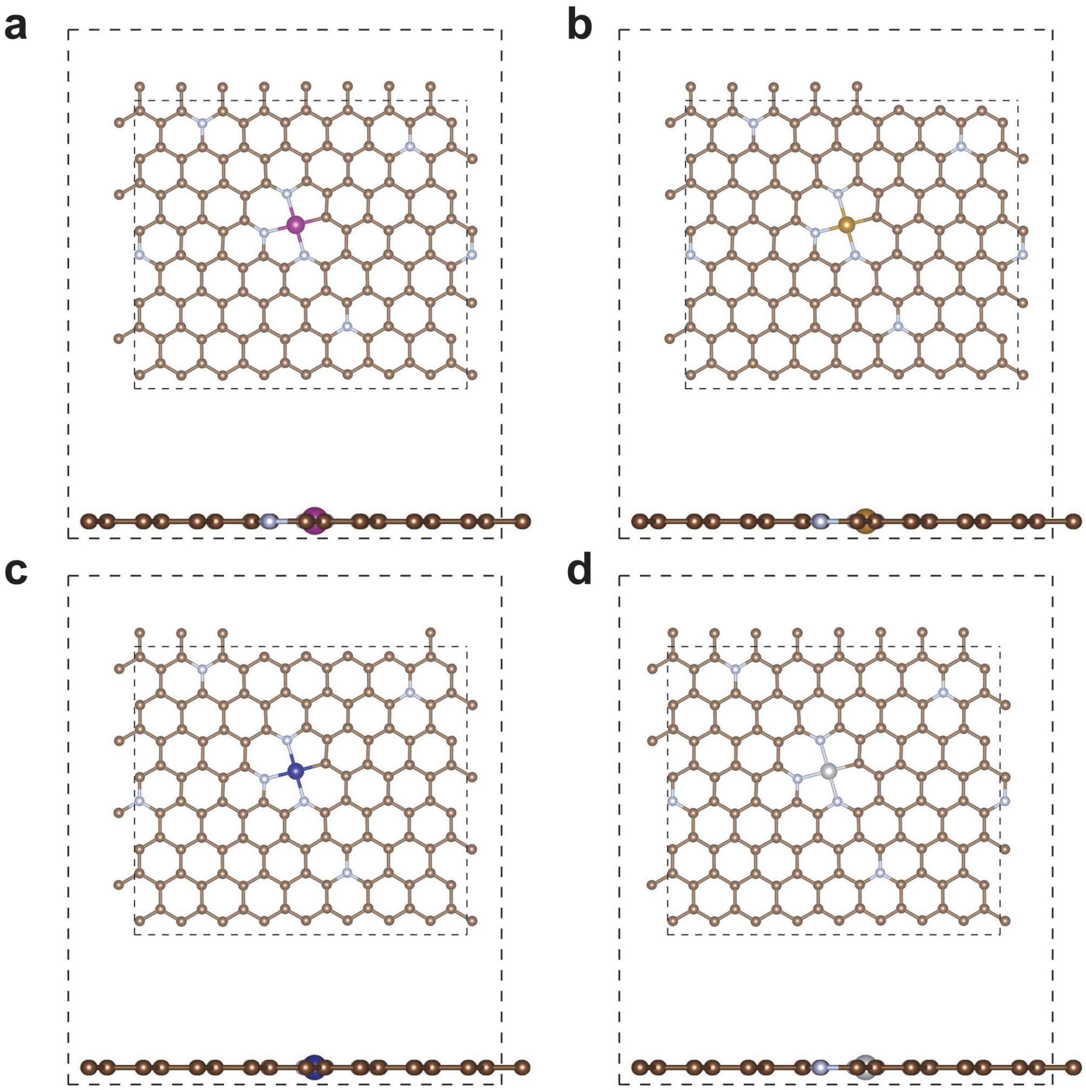

Supplementary Fig. 13. **Theoretical models of the MNC catalysts.** Theoretical models of the MNC catalysts (**a−d** MnNC, FeNC, CoNC, and NiNC). Inset: top view. All lengths are given in Å. The purple, yellow, blue, silver gray, brown, and silver balls denote Mn, Fe, Co, Ni, C, and N atoms, respectively.

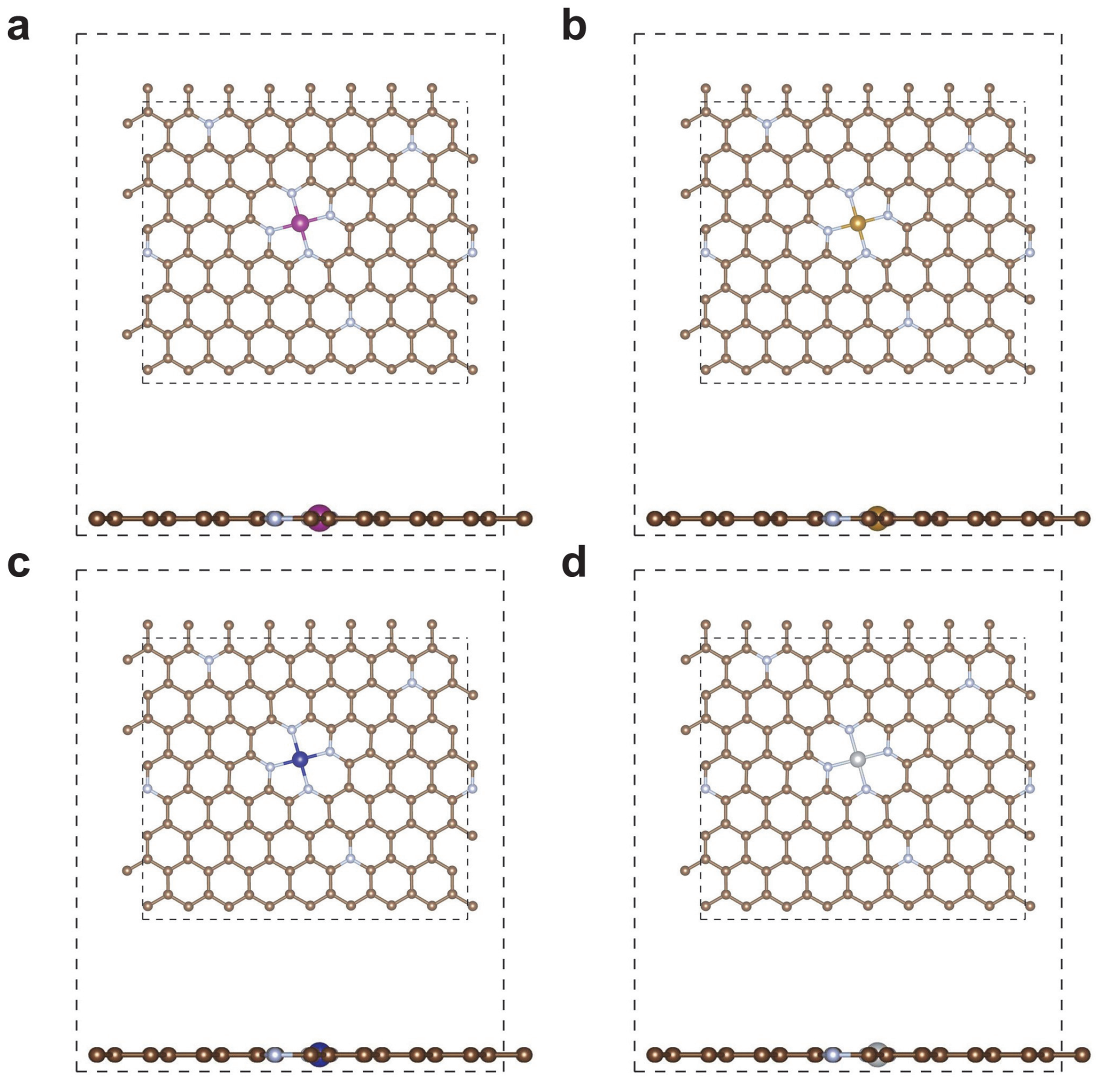

Supplementary Fig. 14. **Theoretical models of the M_1-N_4 units.** Theoretical models of the M_1-N_4 units (**a−d** Mn_1-N_4, Fe_1-N_4, Co_1-N_4, and Ni_1-N_4). Inset: top view. All lengths are given in Å. The purple, yellow, blue, silver gray, brown, and silver balls denote Mn, Fe, Co, Ni, C, and N atoms, respectively.

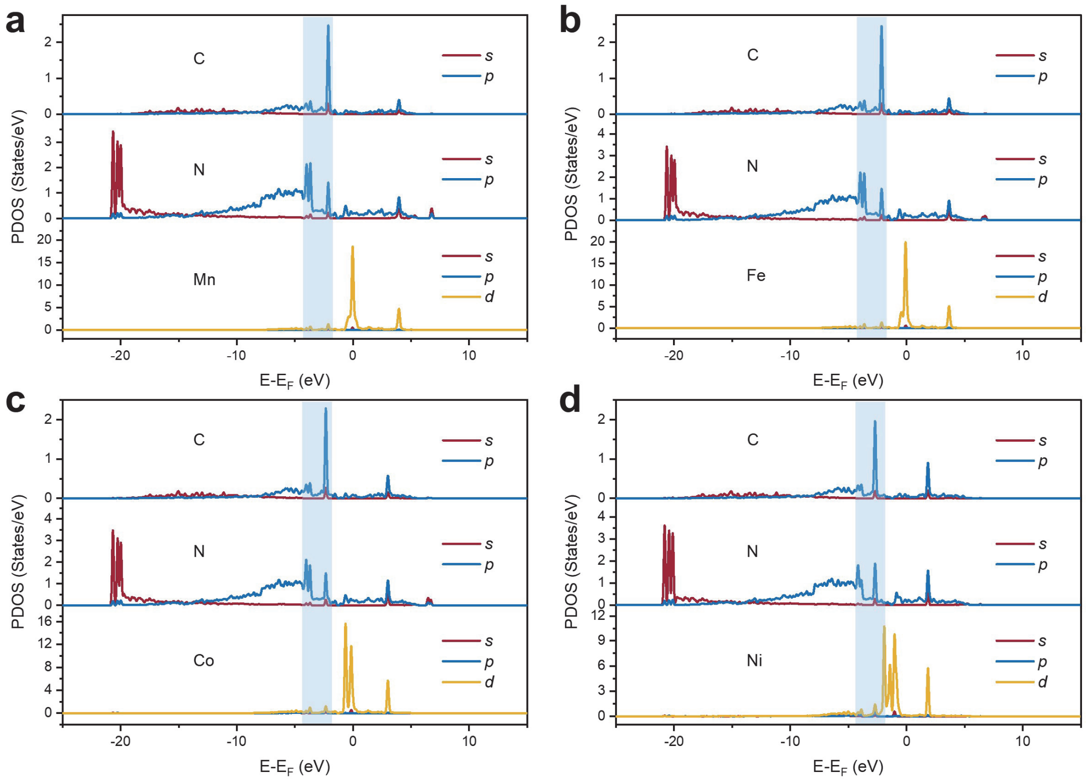

Supplementary Fig. 15. **Partially density of states.** Partially density of states (PDOS) of the MNC catalysts (**a–d** MnNC, FeNC, CoNC, and NiNC).

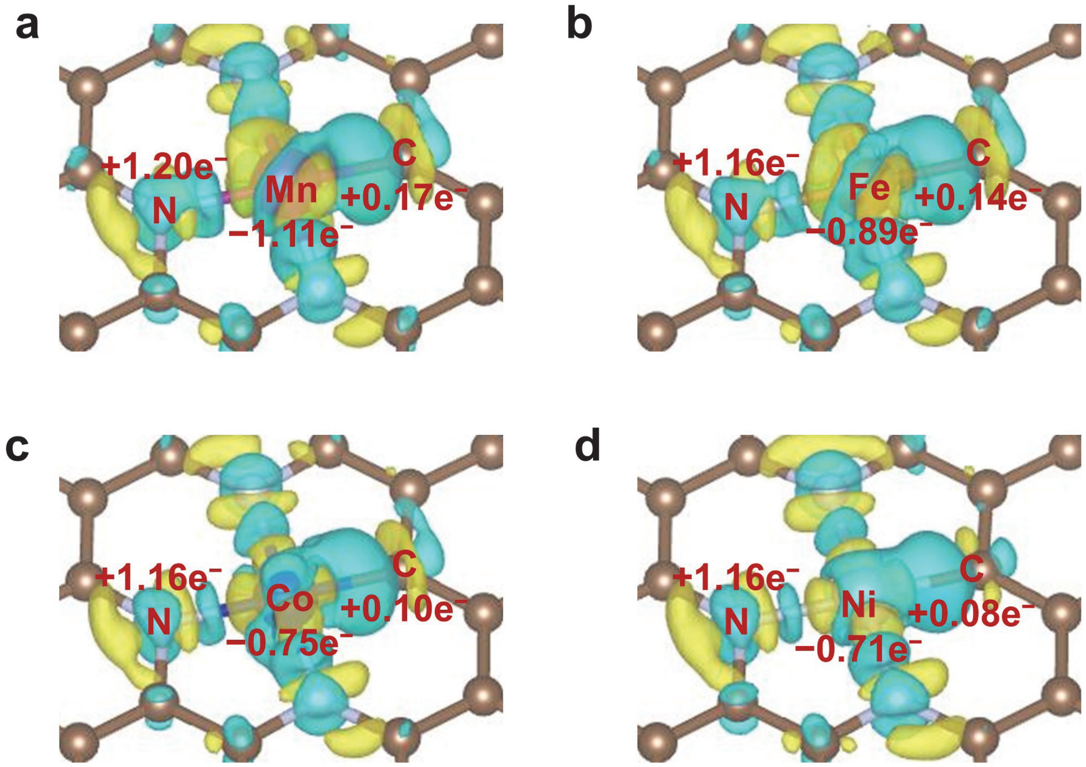

Supplementary Fig. 16. **The charge distribution of the M_1-N_3C_1 units.** Charge difference isosurfaces and Bader charge of the M_1-N_3C_1 units (**a−d** Mn_1-N_3C_1, Fe_1-N_3C_1, Co_1-N_3C_1, and Ni_1-N_3C_1). Isosurfaces level = 0.005. All lengths are given in Å. The purple, yellow, blue, silver gray, brown, and silver balls denote Mn, Fe, Co, Ni, C, and N atoms, respectively. The blue and yellow isosurfaces represent charge accumulation and depletion in the space, respectively.

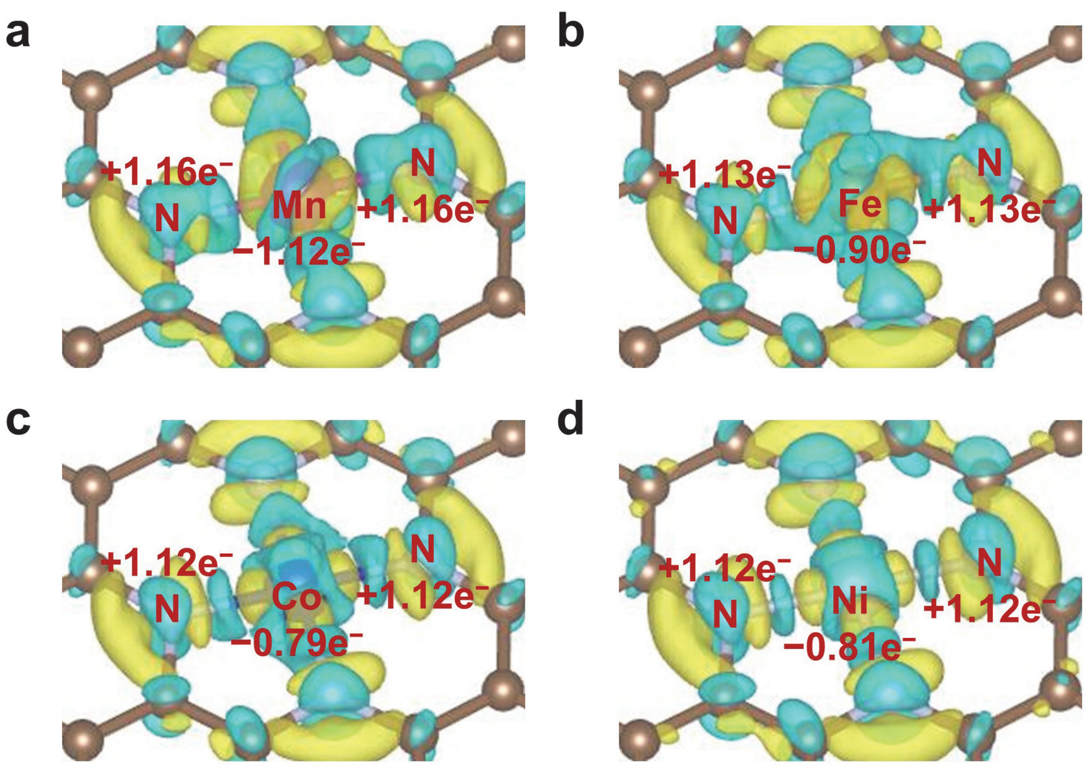

Supplementary Fig. 17. **The charge distribution of the M_1-N_4 units.** Charge difference isosurfaces and Bader charge of the M_1-N_4 units (**a−d** Mn_1-N_4, Fe_1-N_4, Co_1-N_4, and Ni_1-N_4). Isosurfaces level = 0.005. All lengths are given in Å. The purple, yellow, blue, silver gray, brown, and silver balls denote Mn, Fe, Co, Ni, C, and N atoms, respectively. The blue and yellow isosurfaces represent charge accumulation and depletion in the space, respectively.

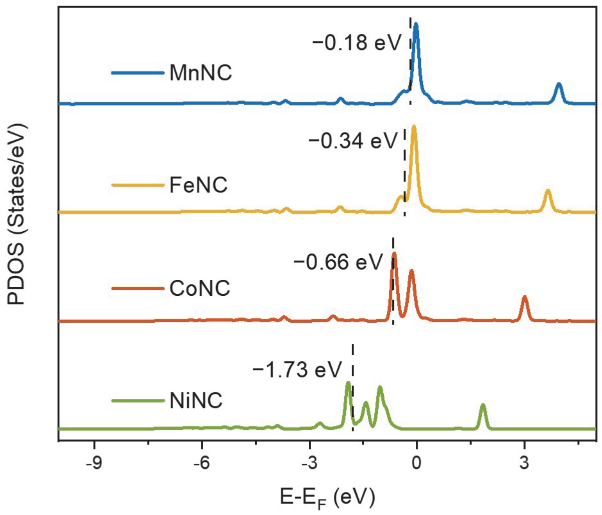

Supplementary Fig. 18. **The *d*-band centers.** The *d*-band centers of the MNC catalysts.

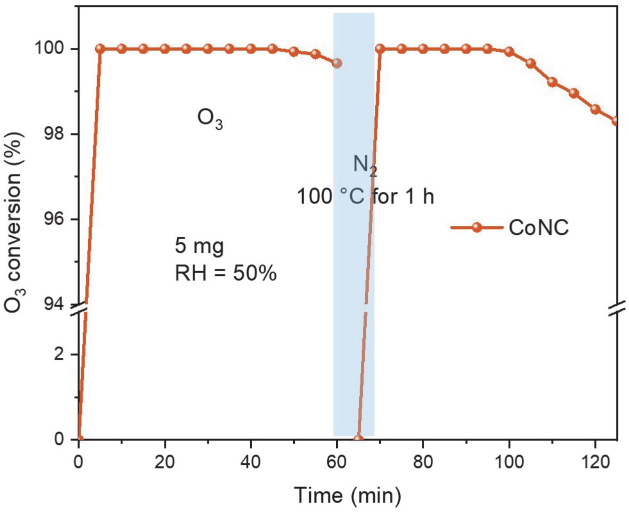

Supplementary Fig. 19. **The regeneration ability of the CoNC.** Catalytic ozone decomposition by continuous use of the CoNC regenerated by annealing.

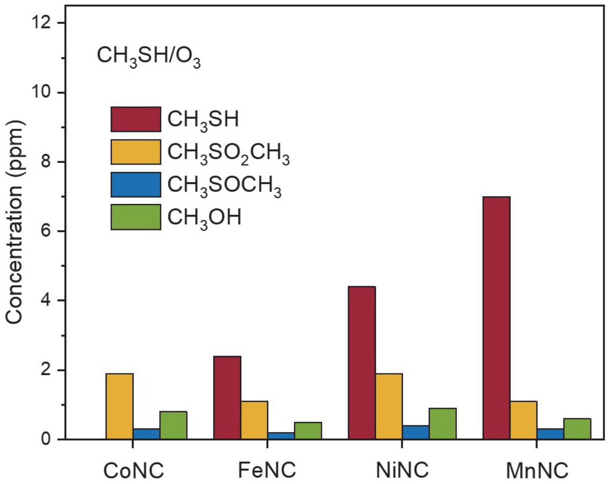

Supplementary Fig. 20. **Exhaust gas concentration after catalytic ozonation.** The concentrations of methyl mercaptan and typical intermediates in the outlet gases of the MNC catalysts after the catalytic ozonation reactions for 60 min determined by proton transfer reaction time-of-flight mass spectrometry (PTR-TOF-MS).

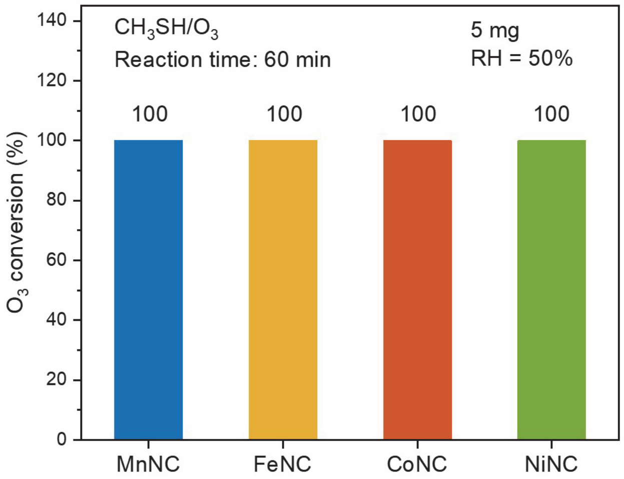

Supplementary Fig. 21. **Ozone conversion efficiency in catalytic ozonation.** Catalytic ozonation for methyl mercaptan degradation tests over the MNC catalysts.

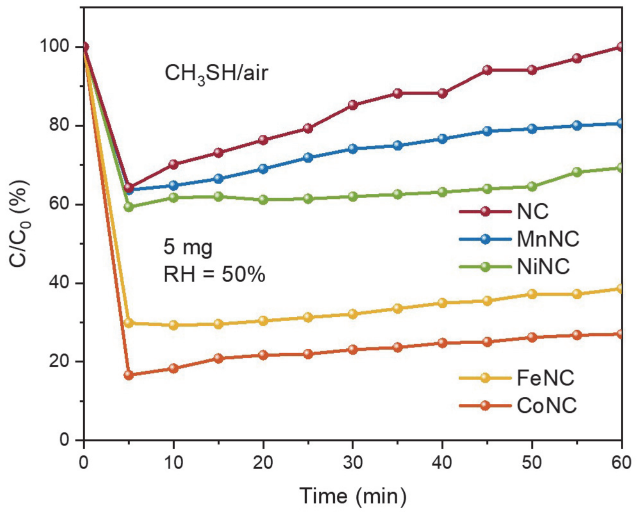

Supplementary Fig. 22. **Methyl mercaptan dynamic degradation tests in the air.** Methyl mercaptan dynamic degradation tests over the NC and MNC catalysts in the air.

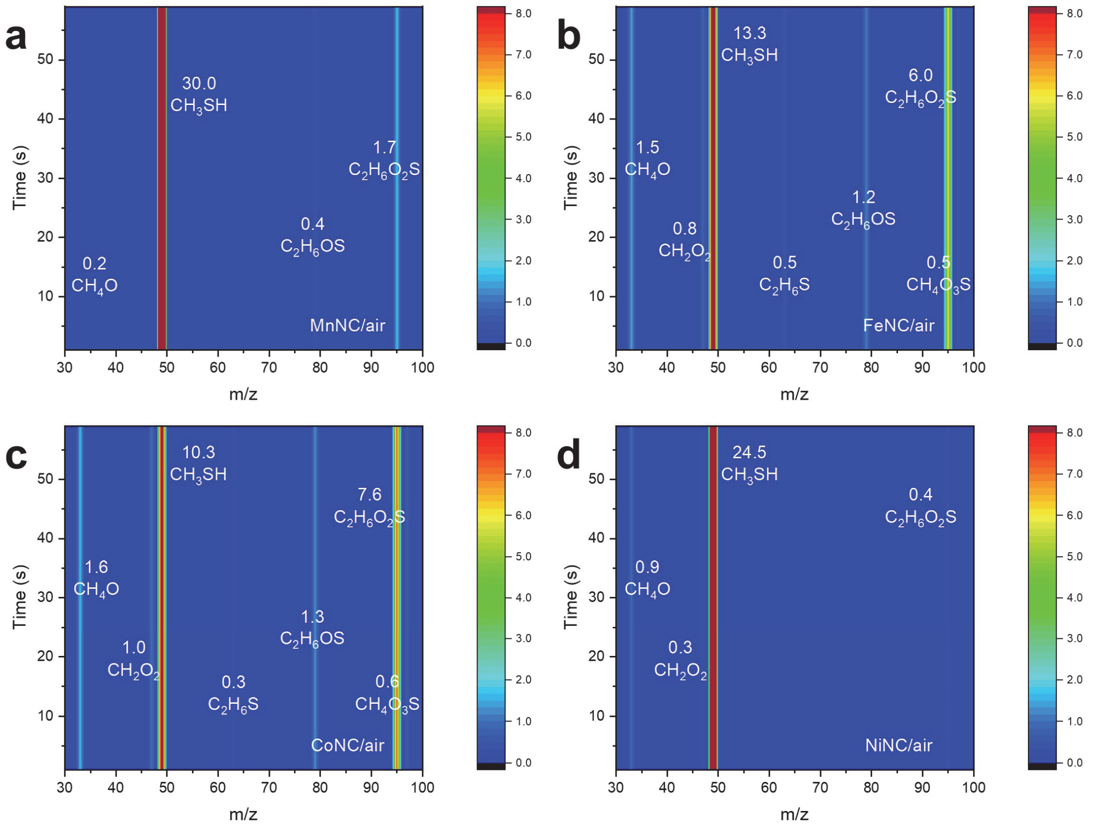

Supplementary Fig. 23. **Exhaust gas concentration after methyl mercaptan dynamic degradation tests in the air.** The concentrations of methyl mercaptan and typical intermediates in the outlet gases of the MNC catalysts after the methyl mercaptan dynamic degradation tests in the air for 60 min determined by proton transfer reaction time-of-flight mass spectrometry (PTR-TOF-MS) (**a–d** MnNC, FeNC, CoNC, and NiNC).

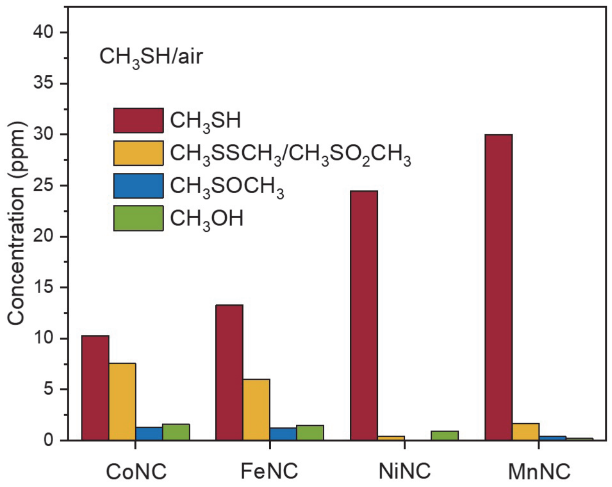

Supplementary Fig. 24. **Exhaust gas concentration after methyl mercaptan dynamic degradation tests in the air.** The concentrations of methyl mercaptan and typical intermediates in the outlet gases of the MNC catalysts after the methyl mercaptan dynamic degradation tests in the air for 60 min determined by proton transfer reaction time-of-flight mass spectrometry (PTR-TOF-MS).

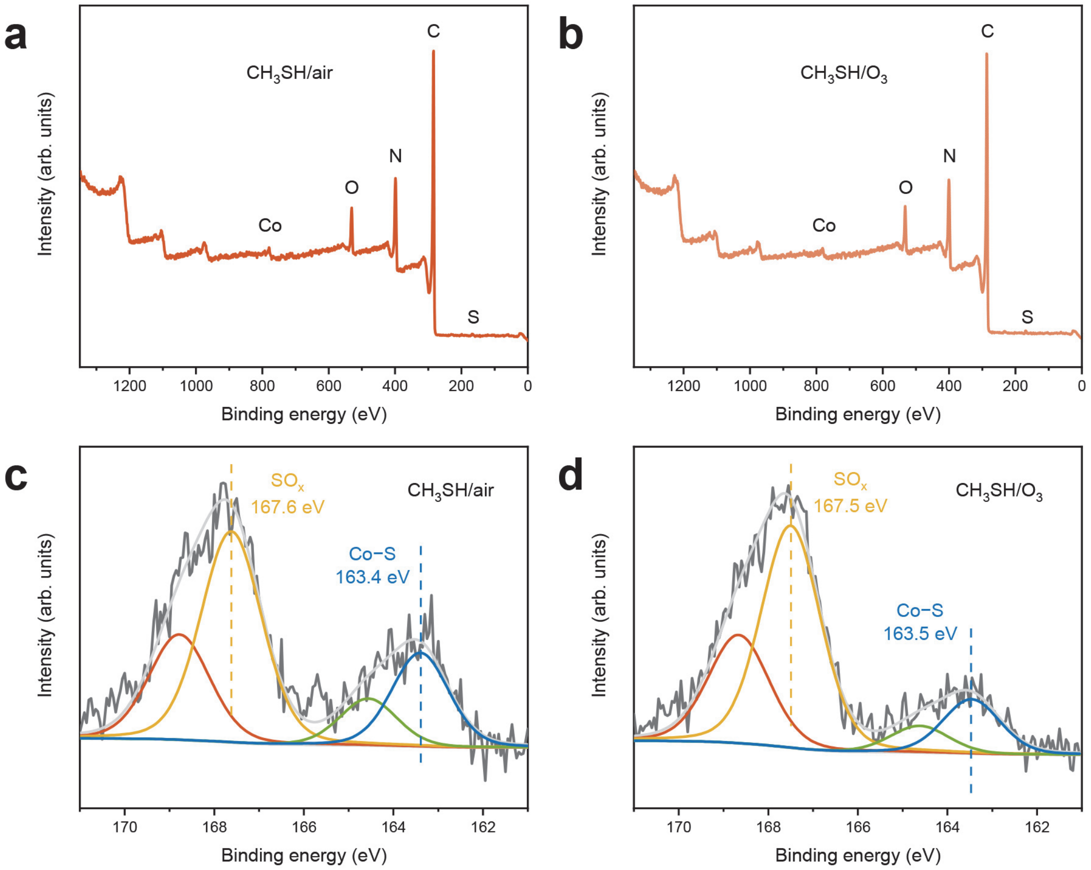

Supplementary Fig. 25. **Surface composition of the reacted CoNC.** X-ray photoelectron spectroscopy (XPS) of the CoNC in different states (**a** survey spectrum of the CoNC after the methyl mercaptan dynamic degradation test in the air for 60 min. **b** survey spectrum of the CoNC after the catalytic ozonation for methyl mercaptan degradation test for 60 min. **c** the S 2*p* spectrum of the CoNC after the methyl mercaptan dynamic degradation test in the air for 60 min. **d** the S 2*p* spectrum of the CoNC after the catalytic ozonation for methyl mercaptan degradation test for 60 min.).

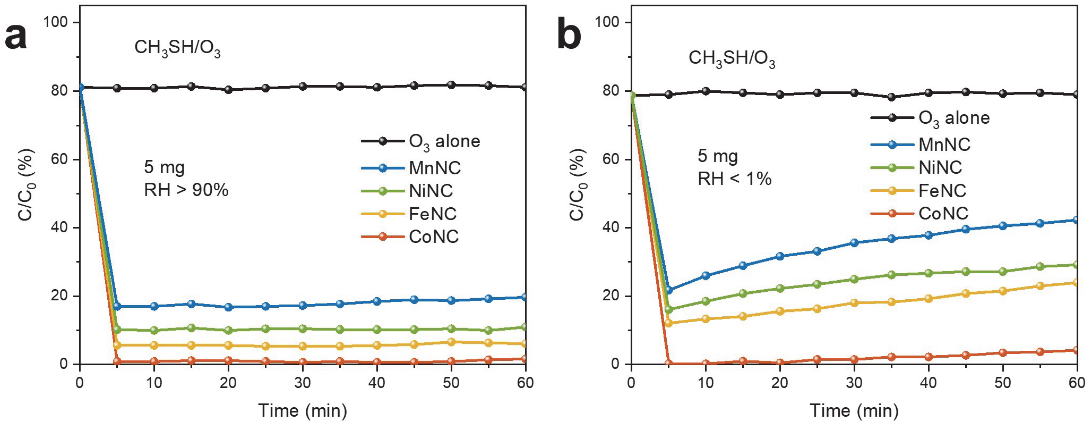

Supplementary Fig. 26. **Catalytic ozonation tests under different relative humidity conditions.** Catalytic ozonation for methyl mercaptan degradation tests over the MNC catalysts under different relative humidity (RH) conditions (**a** RH > 90%. **b** RH < 1%.).

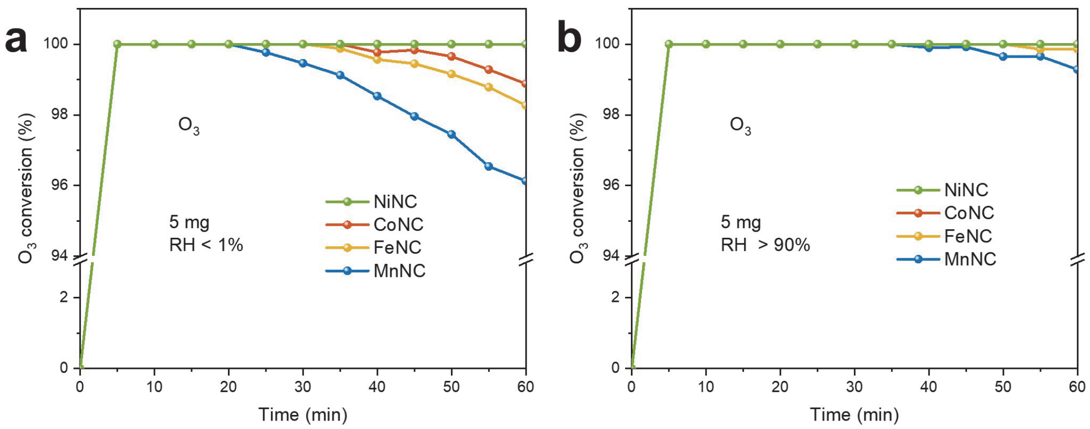

Supplementary Fig. 27. **Catalytic ozone decomposition tests under different relative humidity conditions.** Catalytic ozone decomposition tests over the MNC catalysts under different relative humidity (RH) conditions (**a** RH < 1%. **b** RH > 90%.).

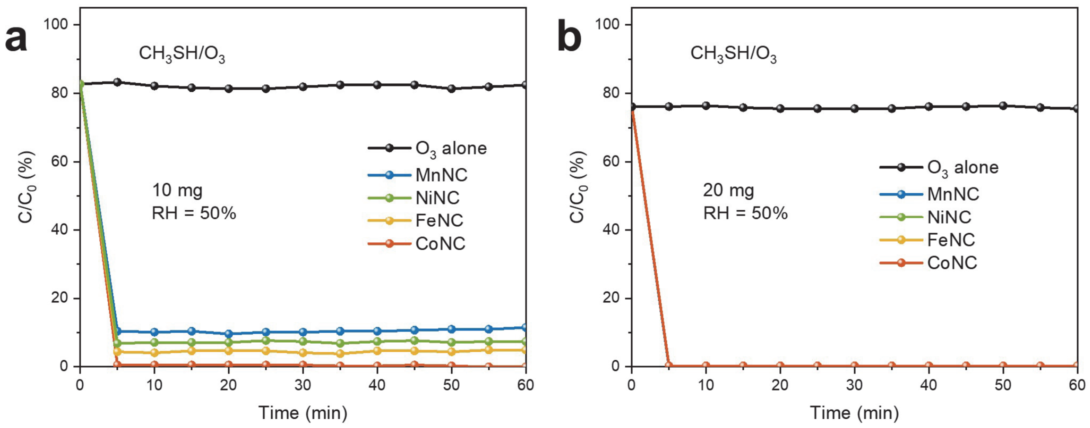

Supplementary Fig. 28. **Catalytic ozonation tests under different catalyst dosages.** Catalytic ozonation for methyl mercaptan degradation tests over the MNC catalysts with different dosages (**a** 10 mg. **b** 20 mg.).

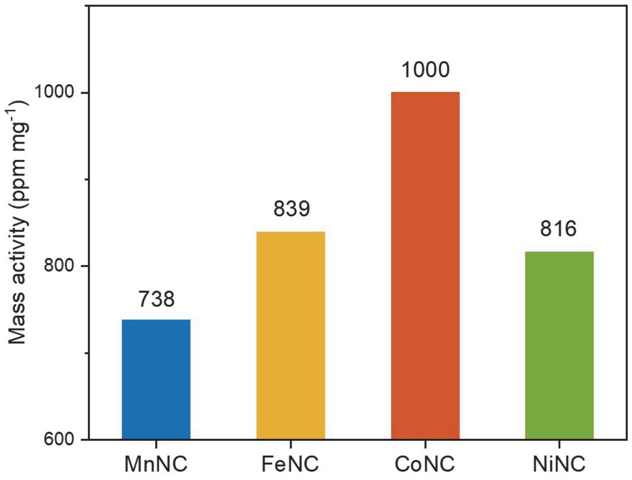

Supplementary Fig. 29. **The mass activity in the tests of catalytic ozonation.** The mass activity of the MNC catalysts in the tests of catalytic ozonation for methyl mercaptan degradation.

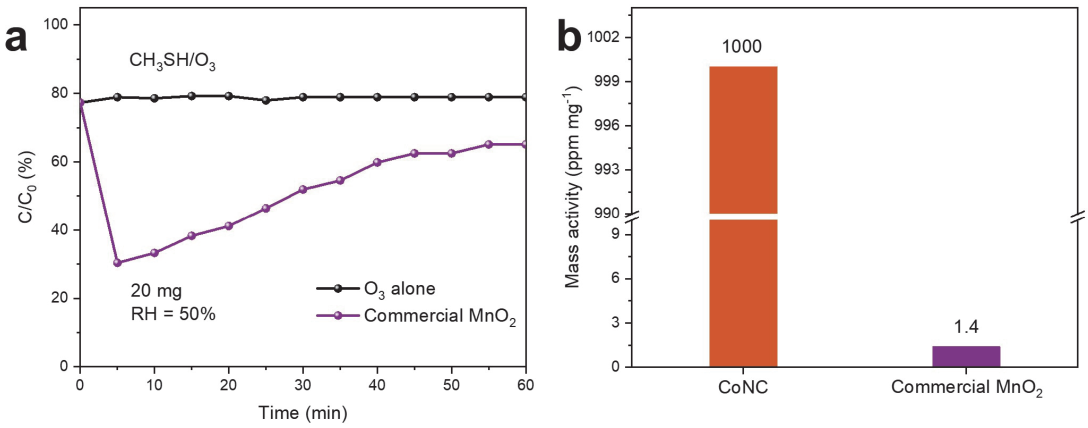

Supplementary Fig. 30. **Catalytic ozonation tests.** Catalytic ozonation for methyl mercaptan degradation tests over commercial manganese dioxide (**a**) and the corresponding mass activity (**b**).

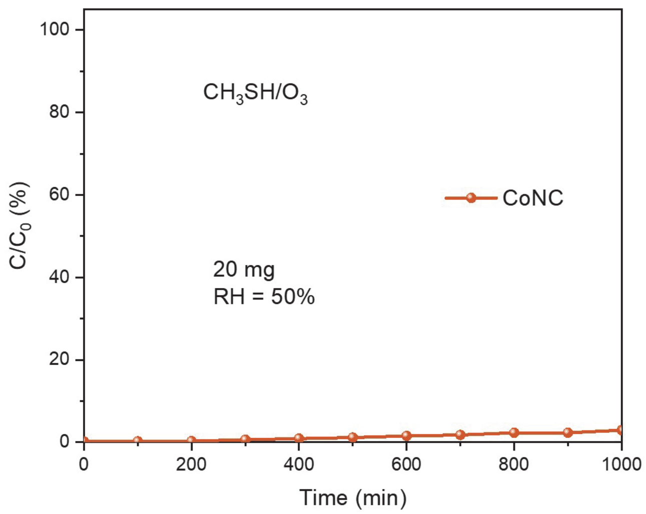

Supplementary Fig. 31. **Long-term test of catalytic ozonation.** Long-term test of catalytic ozonation for methyl mercaptan degradation over the CoNC.

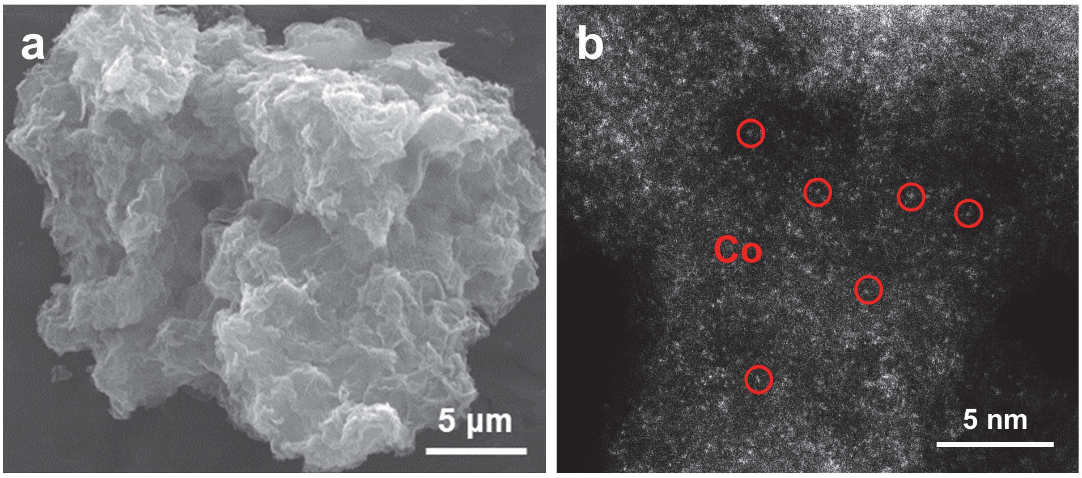

Supplementary Fig. 32. **Morphology of the used CoNC.** Scanning electron microscope (**a** SEM) and aberration-corrected high-angle annular dark-field scanning transmission electron microscope (**b** AC HAADF-STEM) images of the used CoNC catalyst.

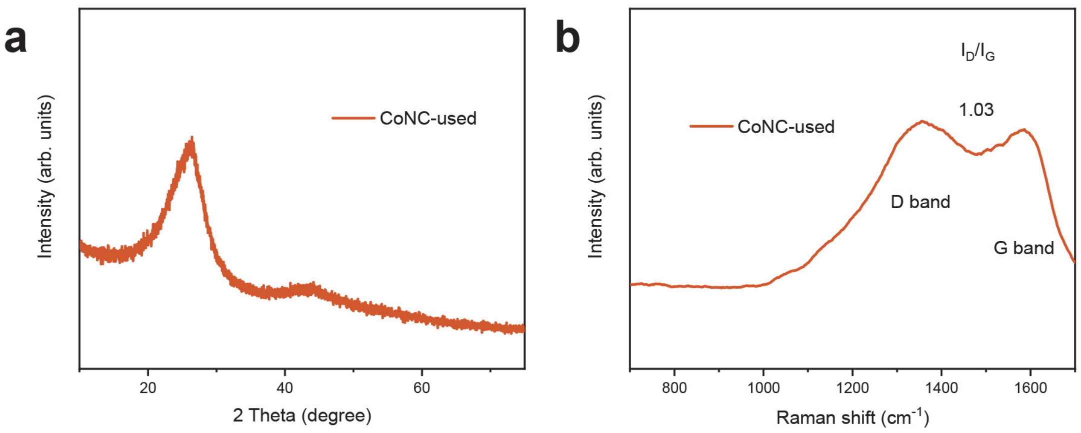

Supplementary Fig. 33. **Composition of the used CoNC. a** Powder X-ray diffraction (XRD) pattern of the used CoNC catalyst. **b** Raman spectrum of the used CoNC catalyst.

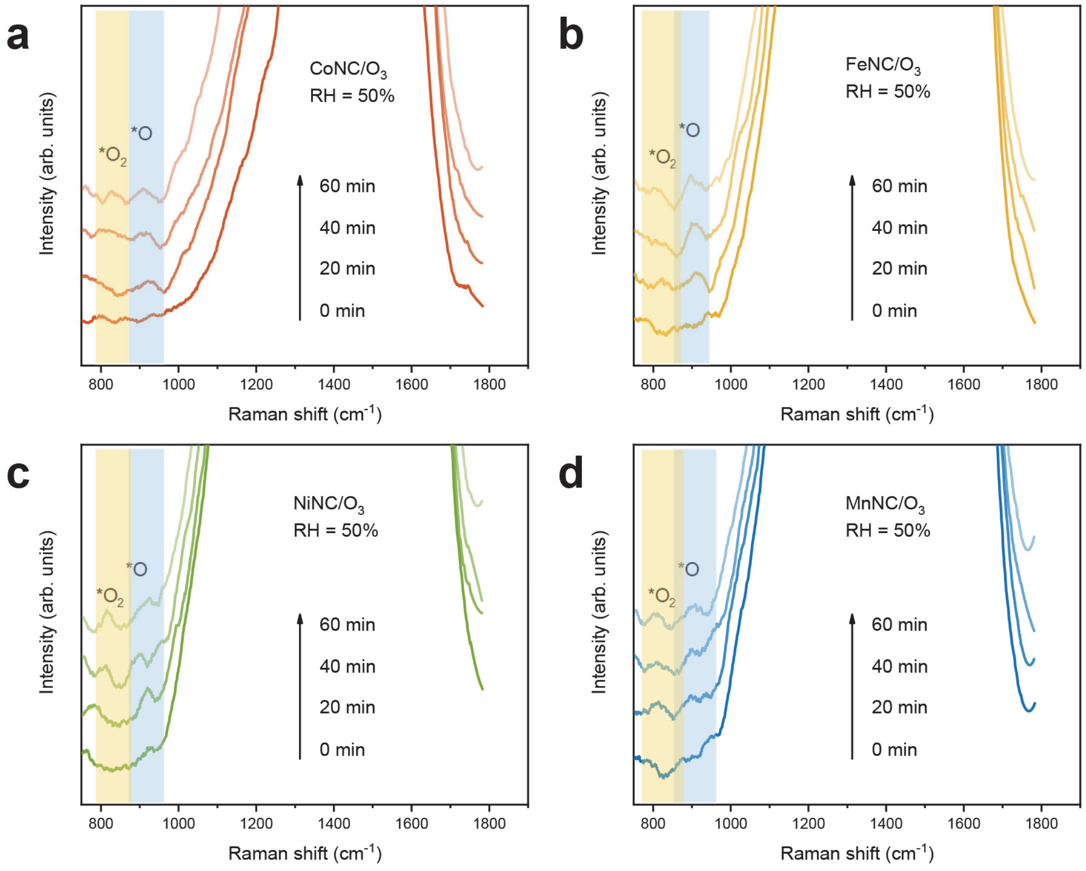

Supplementary Fig. 34. **Reaction of ozone on the surface of the MNC catalysts.** In situ Raman spectra of the MNC catalysts in the ozone atmosphere (**a−d** MnNC, FeNC, CoNC, and NiNC).

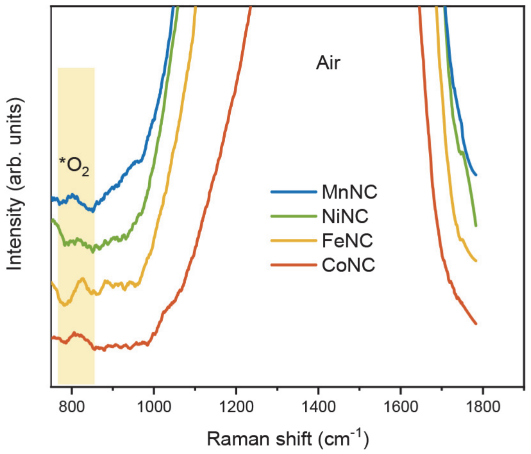

Supplementary Fig. 35. **Complexes on the surface of the MNC catalysts in the air.**

Raman spectra of the MNC catalysts in the air.

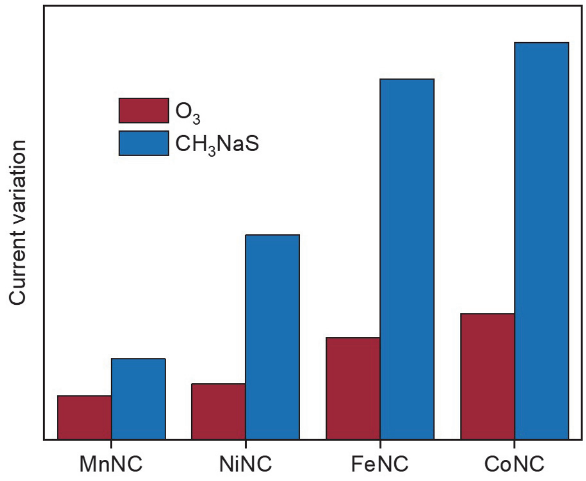

Supplementary Fig. 36. **Reactivity of MNC catalyst surface complexes.** The corresponding current variation of the chronoamperometry curves on the MNC catalysts.

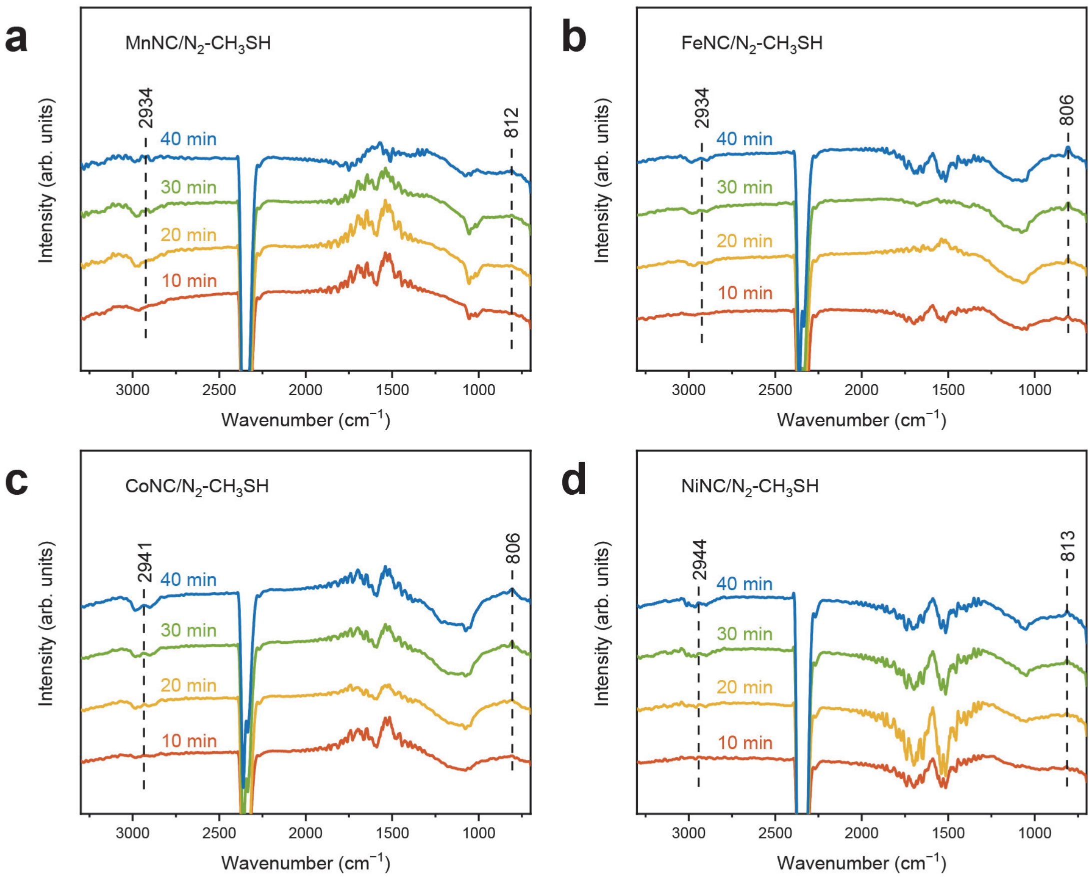

Supplementary Fig. 37. **Reaction process of methyl mercaptan on the surface of the MNC catalysts.** In situ diffuse reflectance infrared Fourier transform (DRIFT) spectroscopy of the MnNC (**a**), FeNC (**b**), CoNC (**c**), and NiNC (**d**) in the CH_3SH/N_2 atmosphere.

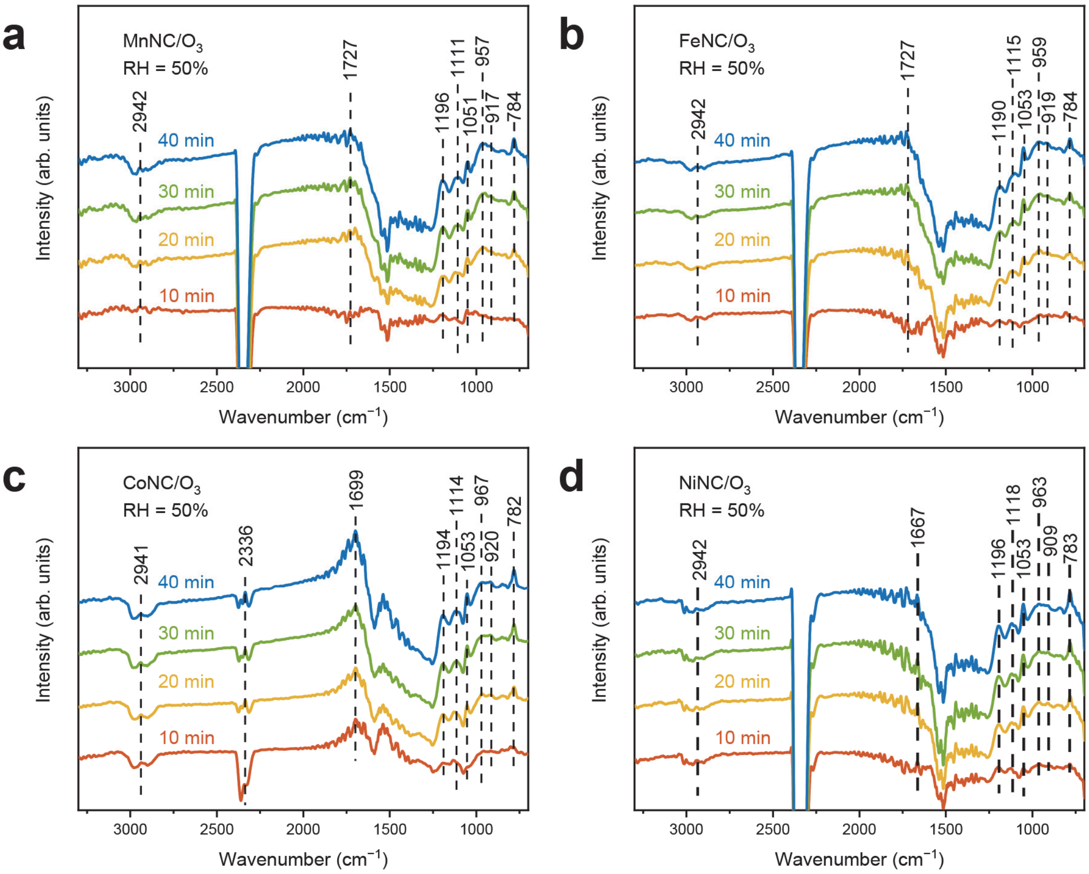

Supplementary Fig. 38. **Reaction process of catalytic ozonation.** In situ diffuse reflectance infrared Fourier transform (DRIFT) spectroscopy of the catalytic ozonation processes over the MnNC (**a**), FeNC (**b**), CoNC (**c**), and NiNC (**d**).

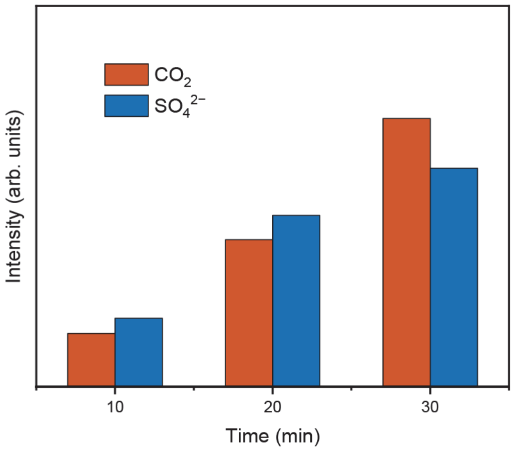

Supplementary Fig. 39. **Species accumulation on the CoNC.** The concentrations of carbon dioxide and sulfate of the catalytic ozonation process over the CoNC (Fig. 4f).

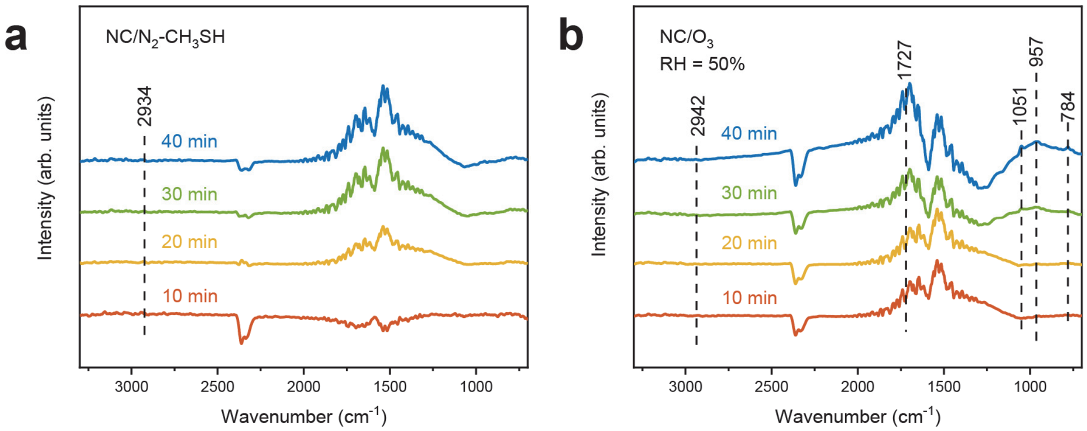

Supplementary Fig. 40. **Reaction process of catalytic ozonation on the surface of the NC. a** In situ diffuse reflectance infrared Fourier transform (DRIFT) spectroscopy of the NC in the CH_3SH/N_2 atmosphere. **b** In situ DRIFT spectroscopy of the catalytic ozonation processes over the NC.

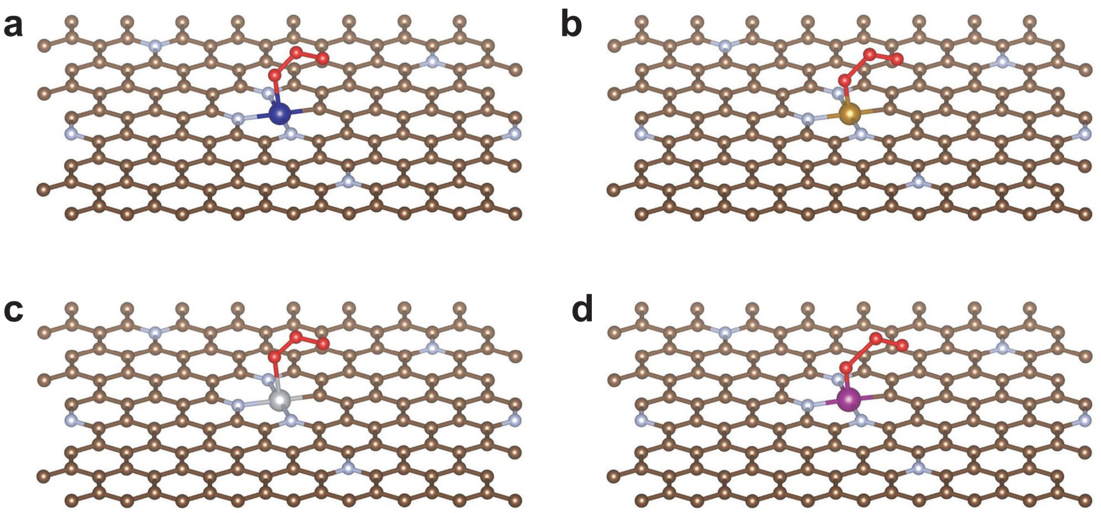

Supplementary Fig. 41. **Reactions between ozone molecules and single metal atoms.** The optimized adsorption of ozone molecule on the MNC coordination structures (**a−d** CoNC, FeNC, NiNC, and MnNC). All lengths are given in Å. The purple, yellow, blue, silver gray, brown, red, and silver balls denote Mn, Fe, Co, Ni, C, O, and N atoms, respectively.

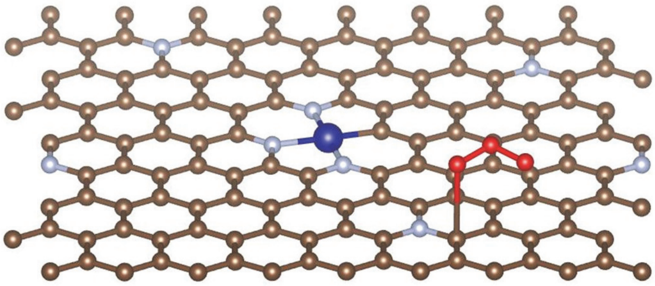

Supplementary Fig. 42. **Reaction between ozone molecule and C atom.** The optimized adsorption of ozone molecule on C atoms. All lengths are given in Å. The blue, brown, red, and silver balls denote Co, C, O, and N atoms, respectively.

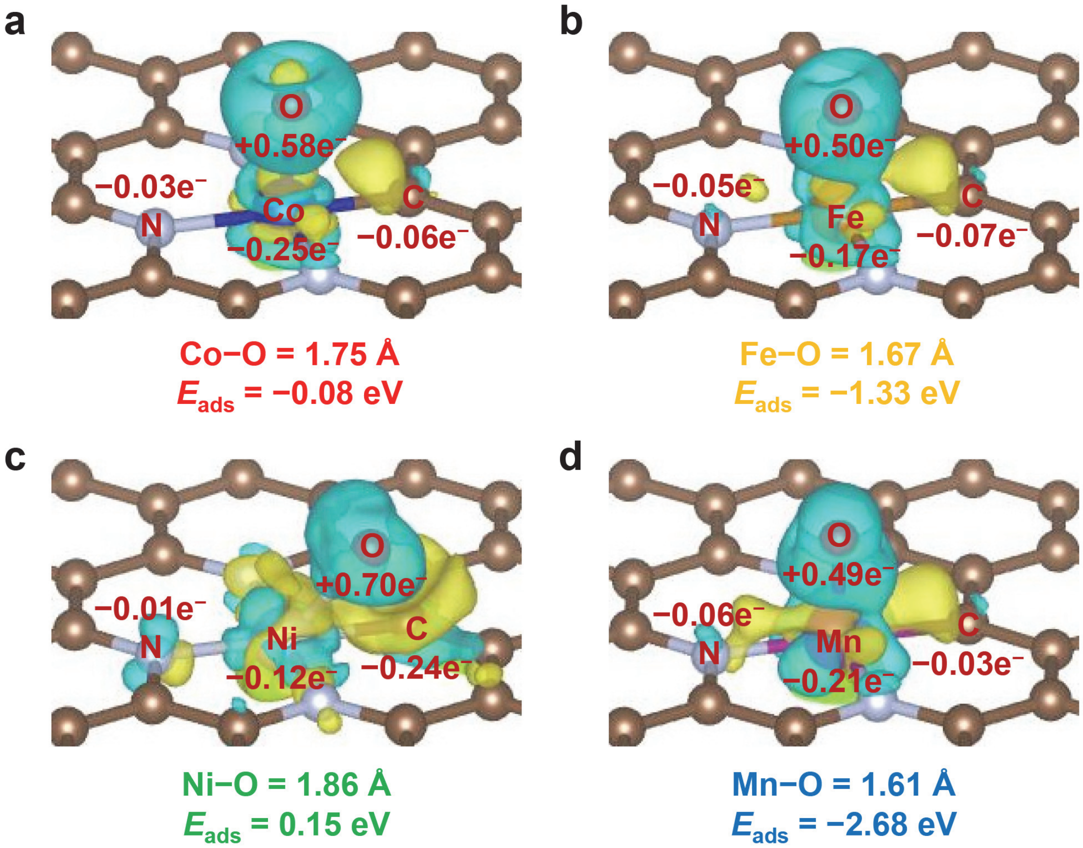

Supplementary Fig. 43. **Theoretical models of the M−*O complexes.** Charge difference isosurfaces, adsorption energies (E_{ads}), and Bader charge of the M−*O complexes (**a−d** Co−*O, Fe−*O, Ni−*O, and Mn−*O). Isosurfaces level = 0.005. All lengths are given in Å. The purple, yellow, blue, silver gray, brown, red, and silver balls denote Mn, Fe, Co, Ni, C, O, and N atoms, respectively. The blue and yellow isosurfaces represent charge accumulation and depletion in the space, respectively.

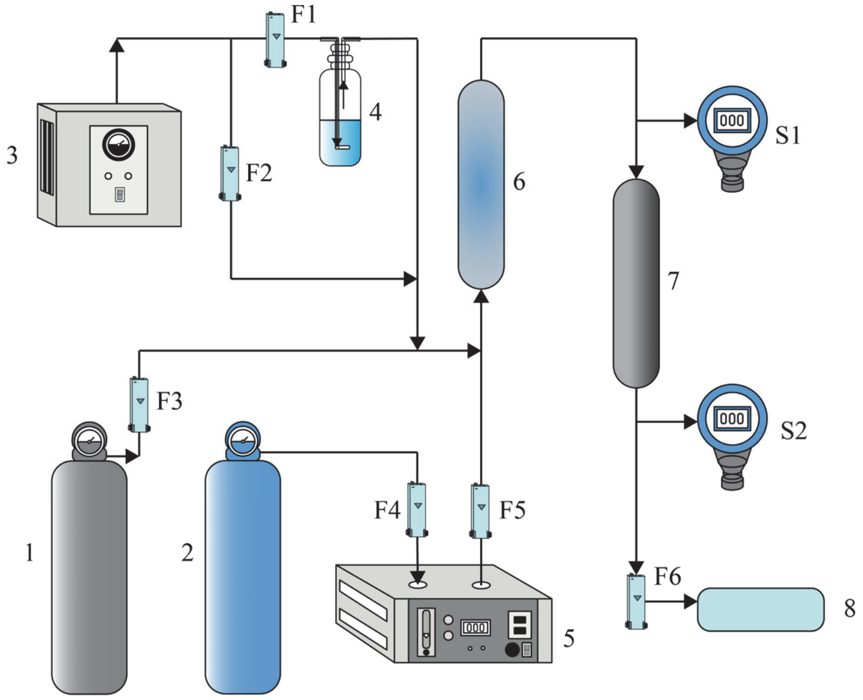

Supplementary Fig. 44. **Schematic diagram of the experimental device.** Schematic diagram of the experimental device for catalytic activity testing. (1. Methyl mercaptan gas source; 2. Oxygen gas source; 3. Air generator; 4. Humidity regulating bottle; 5. Ozone generator; 6. Mixing bottle; 7. Stainless tube reactor; 8. Exhaust gas absorption bottle; F1−F6. Mass flowmeters; S1−S2. Methyl mercaptan/ozone sensor)

Supplementary Tables

Supplementary Table 1. Textural properties of the MNC catalysts.

Samples	Content (wt.%)	BET surface area (m^2 g^{-1})	Pore volume (cm^3 g^{-1})
MnNC	0.99	179.48	0.37
FeNC	1.04	237.12	0.44
CoNC	1.00	179.57	0.39
NiNC	1.00	204.65	0.48

Supplementary Table 2. The elemental content and state percentages of the MNC catalysts.

Samples	Metal content (at.%)	O content (at.%)	N						Metal−N/Metal
			Content (at.%)	Pyridinic	Metal−N	Pyrrolic	Graphitic	NO_x	
MnNC	0.28	4.47	16.71	37.52	5.9	6.42	34.44	15.72	3.52
FeNC	0.3	5.95	15.29	35.03	7.04	2.38	41.91	13.64	3.58
CoNC	0.32	5.99	16.32	37.19	6.18	5.27	36.25	15.11	3.15
NiNC	0.33	4.55	16.15	33.79	6.01	4.87	39.46	15.87	2.94

Supplementary Table 3. Extended X-ray absorption fine structure fitting data for Co center at the Co K-edge.

Sample	Shell	CN	R (Å)	σ^2 *10^{-2} (Å^2)	ΔE_0 (eV)	R factor
CoN_4	Co−N	3.92±0.76	2.07±0.03	1.27±0.52	1.72±2.22	0.022
$Co{-}N_3C_1$	Co−N	3*	2.01±0.09	0.66±0.83	0.75±6.07	0.007
	Co−C	1*	2.17±0.12	0.19±1.34		

CN: coordination numbers; R: bond distance; σ^2: Debye-Waller factors; ΔE_0: the inner potential correction; R factor: goodness of fit.

Supplementary Table 4. Identified conversion products of methyl mercaptan in the air and ozone atmosphere by PTR-TOF-MS.

Entry	Molecule formula	m/z	Possible intermediate
C1	$(CH_2O)H^+$	31	HCOH
C2	$(CH_4O)H^+$	33	CH_3OH
C3	$(CH_2O_2)H^+$	47	HCOOH
C4	$(CH_4S)H^+$	49	CH_3SH
C5	$(C_2H_6S)H^+$	63	CH_3SCH_3
C6	$(CH_4OS)H^+$	65	CH_3SOH
C7	$(C_2H_6SO)H^+$	79	CH_3SOCH_3
C8	$(CH_4SO_2)H^+$	81	CH_3SO_2H
C9	$(C_2H_6S_2/C_2H_6SO_2)H^+$	95	$CH_3SSCH_3/CH_3SO_2CH_3$
C10	$(CH_4O_3S)H^+$	97	CH_3SO_3H

Supplementary Table 5. The elemental content and state percentages of samples.

Sample	S		
	Content (at.%)	Co−S	SO_x
Fresh CoNC	0	/	/
Used CoNC in air	0.40	29.40	70.60
Used CoNC in O_3	0.52	19.10	80.90

Supplementary Table 6. Detected infrared bands over samples.

	Peak position/cm^{-1}	Band attribution
CH_3SH/N_2	2941	CH_3
	806	S−O
O_3	2941	CH_3
	2336	CO_2
	1699	C = O
	1194	SO_4^{2-}
	1114	SO_4^{2-}
	1053	C−OH
	967	SO_3^{2-}
	920	S = O
	782	S−O

Supplementary Table 7. Optimize the adsorption energy corresponding to the structure of the resting point in the molecular adsorption process of the MNC catalysts.

Catalysts	Adsorption energy (eV)					
	O_3	CH_3SH	O_2	H_2O	CO_2	SO_4^{2-}
CoNC	−1.44	−0.45	−1.49	−0.32	−0.21	−0.13
FeNC	−2.27	−1.22	−2.51	−0.37	−0.20	−0.15
NiNC	−1.00	−0.35	−0.63	−0.24	−0.18	−0.15
MnNC	−3.09	−1.39	−2.99	−0.62	−0.59	−0.87
NC	−0.88	/	/	/	/	/

Supplementary Table 8. Optimize the free energy corresponding to the structure of the resting point in the ozone decomposition process of the MNC catalysts.

Reaction coordinates	Energy (eV)			
	MnNC	FeNC	CoNC	NiNC
Bare surface + $2O_3$ (g)	0	0	0	0
$^*O_3 + O_3$ (g)	−3.09	−2.27	−1.44	−1.00
$^*O + O_2$ (g) + O_3 (g)	−3.13	−1.65	−0.50	−0.27
$^*O_2 + 2O_2$ (g)	−2.75	−2.26	−1.23	−0.52
Bare surface + $3O_2$ (g)	0.23	0.26	0.26	0.11
